# P-1658. Distinct Sphingolipid and Glycerophospholipid Signatures in Post-acute sequelae of SARS-CoV-2 infection

**DOI:** 10.1093/ofid/ofaf695.1833

**Published:** 2026-01-11

**Authors:** Shinya Yamamoto, Makoto Kurano, Koh Okamoto, Takeya Tsutsumi, Kyoji Moriya

**Affiliations:** The Univeristy of Tokyo, Tokyo, Tokyo, Japan; The University of Tokyo, Tokyo, Tokyo, Japan; Institute of Science Tokyo, Bunkyo-ku, Tokyo, Japan; The University of Tokyo Hospital, Bunkyo-ku, Tokyo, Japan; The University of Tokyo, Tokyo, Tokyo, Japan

## Abstract

**Background:**

Post-acute sequelae of SARS-CoV-2 infection (PASC) is a multifaceted condition with multiorgan symptoms affecting millions globally. Though its mechanisms remain poorly understood, persistent immunometabolic disruption is suspected. Given the known role of lipidomes in Alzheimer's disease, we aimed to identify lipidomic biomarkers of PASC.

**Figure:**
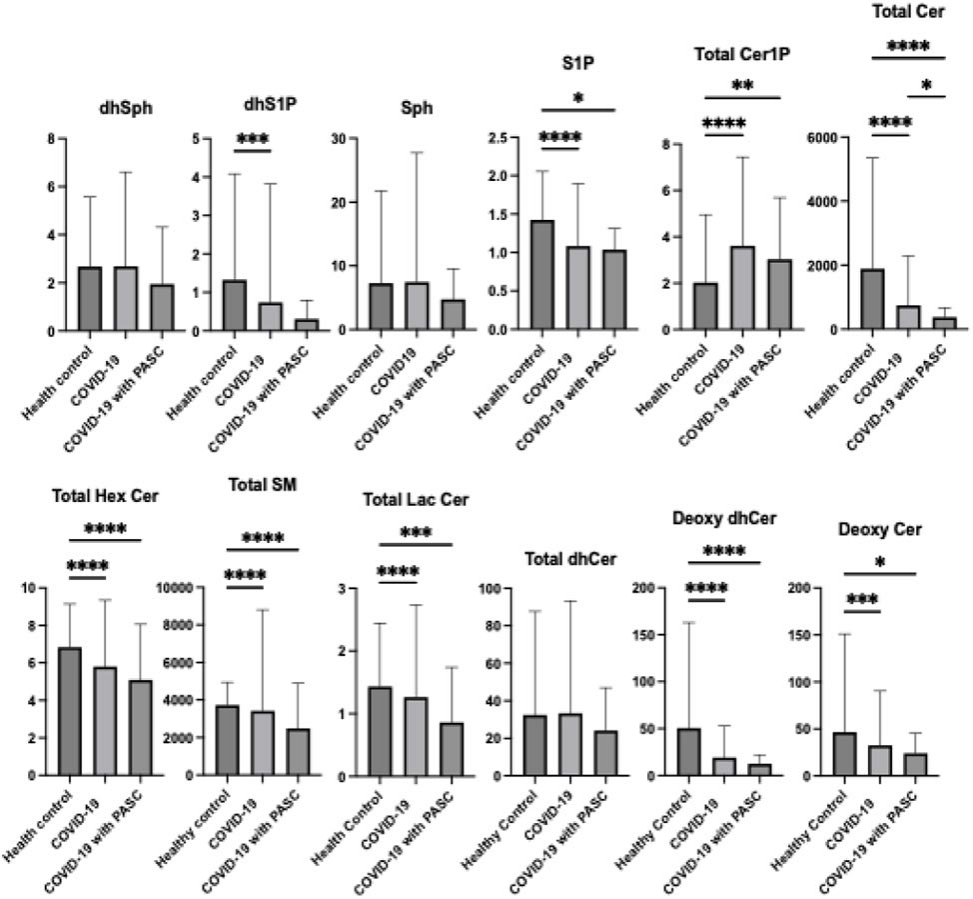
Sphingolipid levels

**Figure:**
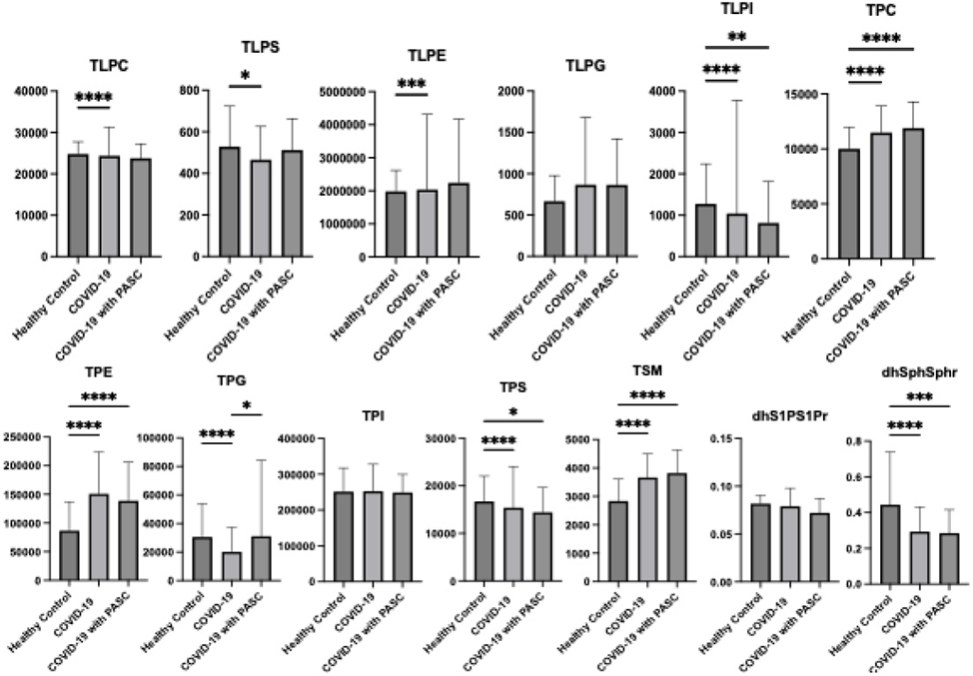
Glycerophospholipid levels.

**Methods:**

We performed targeted lipidomic analysis using a liquid chromatography tandem mass spectrometry on plasma samples from healthy controls and COVID-19 patients hospitalized and followed as outpatient at the University of Tokyo Hospital, 2020-2022. We focused on sphingolipid and glycerophospholipid metabolites, key regulators of cell survival, inflammation, and cytokine responses. Sparse partial least squares-discriminant analysis (sPLS-DA) and Kendall rank correlation were used to identify lipids characteristic of PASC and explore associations with clinical parameters.

**Figure:**
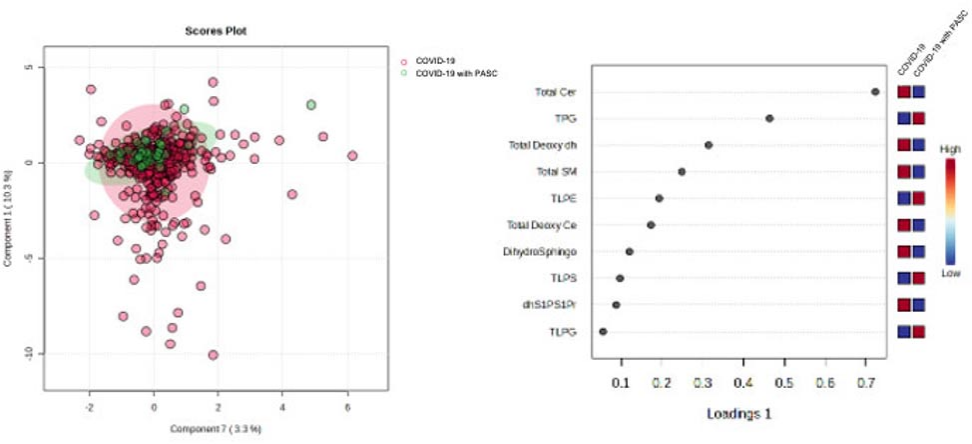
Multivariate analysis of Sphingolipids and Glycerophospholipid levels in COVID-19 PASC, sPLSDA-models.

**Figure:**
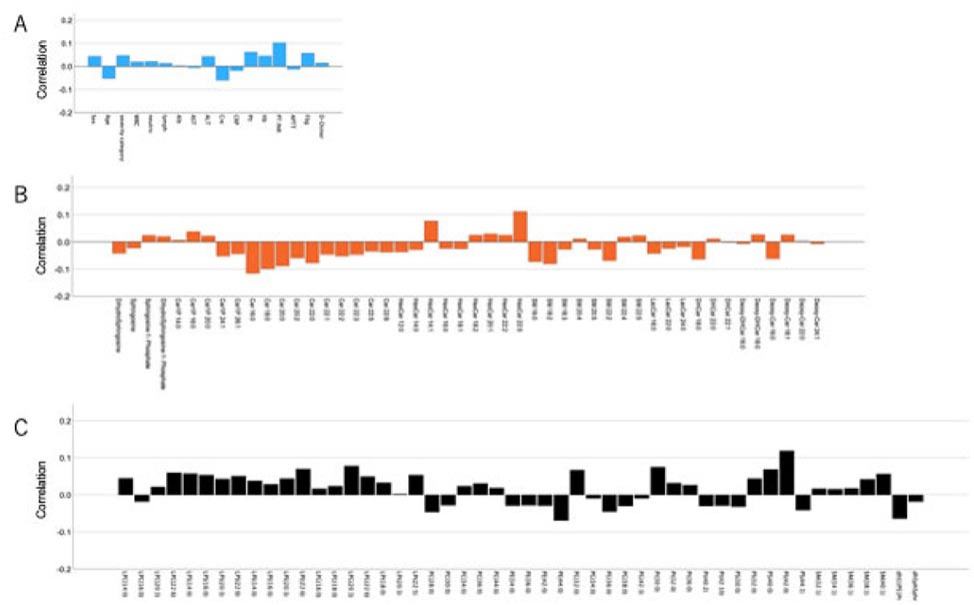
Kendall correlation analysis association with PASC. A: Clinical data, B: Sphingolipid, C: Glycerophospholipid, Columns represent the correlation coefficient

**Results:**

A total of 859 samples from 215 COVID-19 patients and 115 samples from health controls were analyzed. Several sphingolipids, including sphingosine-1-phosphate, dihydro-sphingosine-1-phosphate, ceramide-1-phosphate, ceramide (Cer), sphingomyelin (SM)　was significantly reduced in the COVID-19 (with or without PASC) compared to healthy controls. Notably, Cer levels were significantly lower in PASC compared to COVID-19 without PASC (Figure 1). Glycerophospholipids such as lysophosphatidylcholine (LPC), lysophosphatidylserine (LPS), lysophosphatidylinositol (LPI), and phosphatidylserine (PS) were also reduced. However, phosphatidylglycerol (PG) levels showed a relative rebound in PASC, approaching control levels (Figure 2). sPLS-DA revealed distinct lipidomic clustering among groups, withCer and PG among key discriminators of PASC status (Figure 3). Correlation analysis further highlighted Cer 16:0, Cer 18:0, Cer20:0, Cer 20:2, Cer 22:0, SM 16:0, SM 18:2, and SM 22:2 negativity associated with PASC. Additionally, several glycerophospholipids, LPC 22:6, LPS 14:0, LPE 22:6, LPG 20:3, PE 44:0, PG 32:0, PI 30:0, PS 40:0, and PS 42:0 were associated with PASC (Figure 4).

**Conclusion:**

Distinct sphingolipid and glycerophospholipid alterations characterize PASC and may serve as metabolic biomarkers for this condition.

**Disclosures:**

Makoto Kurano, MD, PhD, Nihon Waters: The present study was a collaborative research project undertaken by The University of Tokyo and Nihon Waters. Koh Okamoto, MD, MS, PhD, Becton, Dickinson and Company: Honoraria|Shionogi: Honoraria|Terumo: Honoraria|Thermo Fisher Scientific: Honoraria

